# Recapitulating Glioma Stem Cell Niches Using 3D Spheroid Models for Glioblastoma Research

**DOI:** 10.3390/bios14110539

**Published:** 2024-11-07

**Authors:** Hyunji Jo, Seulgi Lee, Min-Hyeok Kim, Sungsu Park, Seo-Yeon Lee

**Affiliations:** 1 Department of Metabiohealth, Sungkyunkwan University (SKKU), Suwon 16419, Republic of Korea; guswl5425@g.skku.edu (H.J.); tmfrlckdrjs@g.skku.edu (S.L.); 2 School of Mechanical Engineering, Sungkyunkwan University (SKKU), Suwon 16419, Republic of Korea; mhkim967@g.skku.edu; 3 Department of Quantum Biophysics, Institute of Quantum Biophysics (IQB), Sungkyunkwan University (SKKU), Suwon 16419, Republic of Korea; 4 Biomedical Institute for Convergence at SKKU (BICS), Sungkyunkwan University (SKKU), Suwon 16419, Republic of Korea; 5 Department of Pharmacology, Wonkwang University School of Medicine, Iksan 54538, Republic of Korea; 6 Department of Biomedical Science, Wonkwang University School of Medicine, Iksan 54538, Republic of Korea

**Keywords:** glioblastoma, glioma stem cells, 3D spheroid, heterogeneity, drug resistance

## Abstract

Glioblastoma multiforme (GBM) is among the most aggressive brain cancers, and it contains glioma stem cells (GSCs) that drive tumor initiation, progression, and recurrence. These cells resist conventional therapies, contributing to high recurrence rates in GBM patients. Developing in vitro models that mimic the tumor microenvironment (TME), particularly the GSC niche, is crucial for understanding GBM growth and therapeutic resistance. Three-dimensional (3D) spheroid models provide a more physiologically relevant approach than traditional two-dimensional (2D) cultures, recapitulating key tumor features like hypoxia, cell heterogeneity, and drug resistance. This review examines scaffold-free and scaffold-based methods for generating 3D GBM spheroids, focusing on their applications in studying the cancer stem cell niche. The discussion encompasses methods such as the hanging drop, low-adhesion plates, and magnetic levitation, alongside advancements in embedding spheroids within extracellular matrix-based hydrogels and employing 3D bioprinting to fabricate more intricate tumor models. These 3D culture systems offer substantial potential for enhancing our understanding of GBM biology and devising more effective targeted therapies.

## 1. Introduction

Glioma stem cells (GSCs), a subpopulation with stem cell-like properties, are believed to drive glioblastoma multiforme (GBM) initiation, progression, and recurrence [1,2]. Despite the standard treatment—surgical resection followed by radiotherapy and temozolomide chemotherapy—GBM patients have a median survival of 6 months post-surgery and only 14–16 months with these therapies [3,4,5]. The high recurrence rate is mainly due to the persistence of GSCs, which resist conventional treatments. These cells exhibit self-renewal, differentiation, and tumor-regenerative abilities, contributing to tumor development, progression, and treatment resistance [6,7].

GSCs share key properties with normal stem cells, such as self-renewal and differentiation [8,9]. In vitro, GSCs form multicellular three-dimensional (3D) spheroids under non-adherent conditions [10,11]. These 3D spheroids closely mimic solid tumors in vivo, making them valuable for studying GBM biology [12]. Spheroid cultures enriched with cancer stem cells (CSCs) have also been observed in other cancers, such as murine lung cancer [13].

**Figure 1:**
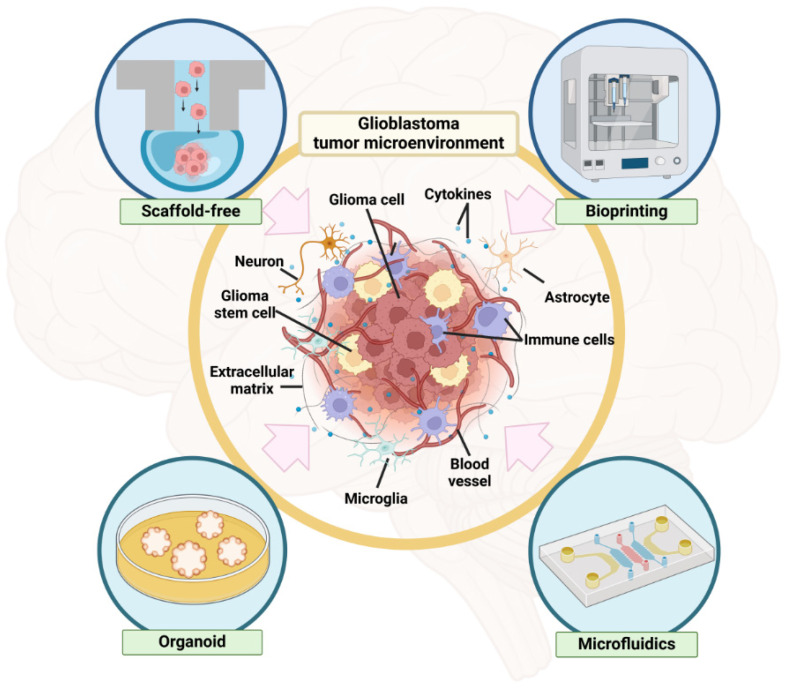
A schematic illustrating the glioblastoma tumor microenvironment (TME) in vitro, consisting of glioma cells, glioma stem cells, stromal cells, and extracellular matrix constructed by several methods: scaffold-free, bioprinting, organoid, and microfluidics. The schematic was created with Biorender.com.

This review, therefore, focuses on 3D spheroid models in GBM research, providing insights into tumor biology and drug resistance mechanisms (Figure 1). Such models are critical for developing more effective targeted therapies.

## 2. GSC Niche in GBM

**Figure 2:**
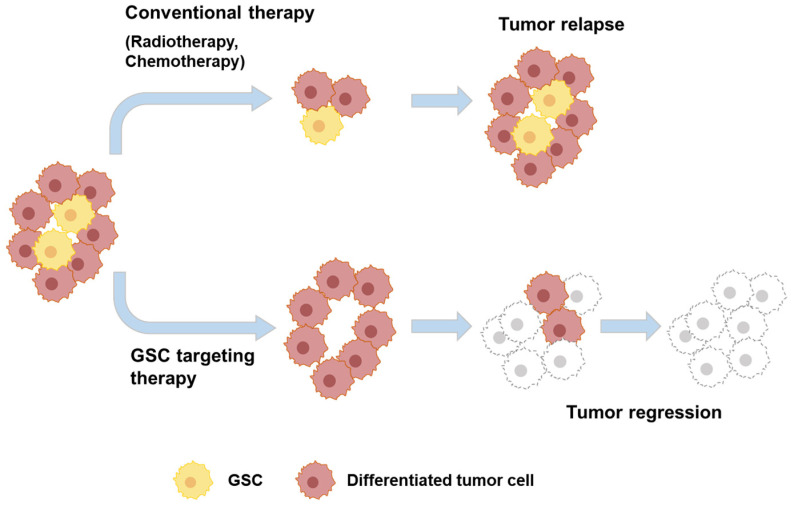
A schematic illustrating the key roles of glioma stem cells (GSCs) within glioblastoma multiforme (GBM) for promoting tumor development, progression, and treatment resistance. GSCs drive these processes by facilitating self-renewal, differentiation, and proliferation. This figure was drawn based on the literature [14].

CSCs are highly tumorigenic and responsible for driving tumor progression, recurrence following conventional chemo- or radiotherapy, and metastasis [14]. This is attributable to their unique capabilities, including self-renewal, differentiation into diverse cell types within the tumor, and survival of treatments typically targeting rapidly dividing cells. CSCs often exhibit increased resistance to therapies due to their ability to remain in a quiescent state and possess efficient DNA repair mechanisms, allowing them to evade treatment-induced cell death. Furthermore, the enhanced plasticity of CSCs enables them to adapt to various microenvironmental cues, promoting metastatic spread. Given the critical role of CSCs in these processes, they have become a promising target for cancer eradication. To overcome the therapeutic challenges posed by CSCs, increasing attention has focused on their specialized microenvironment, termed niche, which helps maintain their stemness and resistance. Disrupting the CSC niche is, therefore, a theoretically sound strategy to weaken the stem-like properties of CSCs, potentially reducing their ability to drive recurrence and metastasis. GBM CSCs (known as glioma stem cells, GSCs) are located in niches within tumors, and the concept of GSCs has provided new insight into GBM resistance and recurrence (Figure 2).

GSCs are localized in the TME, which is often hypoxic (low oxygen conditions). Unlike normal tissues, tumors continually expand in size, eventually outstripping the host’s vascular supply [15]. Oxygen delivery to the inner regions of the solid tumor mass is reduced due to increased diffusion distances between the vasculature and inner tumor regions. Consequently, solid tumors form new blood vessels through angiogenesis [15]. Additionally, vascular structures within GBM are typically abnormal, forming a disorganized network of leaky and inefficient blood vessels. This abnormal vasculature further limits oxygen supply to cells within the tumor mass, promoting an aggressive phenotype and enhancing resistance to therapies [16]. Similarly, limited nutrient and oxygen availability in vitro constrains the growth of tumor spheroids, capping their size at a few hundred microns in diameter.

Hypoxia significantly influences the maintenance and expansion of GSCs [17]. Hypoxia increases the expression of stem-cell markers, CD133, OCT4, and SOX2 [18,19]. CD133, essential for GSC maintenance, is upregulated by hypoxia in glioblastoma neurospheres [18,20]. Other GSC markers that increase under low oxygen levels in GBM include podoplanin, B lymphoma Mo-MLV insertion region 1 homolog (Bmi-1), and nestin [21]. However, Sox2 expression increases only in multicellular tumor spheroids derived from GBM short-term culture with tumor stem cell properties, suggesting that tumor cell phenotypes associated with stemness and chemoresistance depend on the oxygen tension surrounding the tumor cell and cellular interactions [22,23,24].

## 3. GBM Spheroid Formation Methods to Study GSC Niche

Two-dimensional (2D) culture systems grow cells in a flat monolayer. While simple to use and cost-effective, they do not accurately mimic the complex 3D structure of the GSC [25,26,27,28]. Therefore, constructing tumor spheroids in vitro is essential for creating models that closely resemble the TME, providing insights into cancer biology and therapeutic targets. These methods are categorized into scaffold-free and scaffold-based approaches, each with unique benefits and limitations. Many methods have not been applied to recapitulate the GSC niche in GBM, but they hold future potential.

### 3.1. Scaffold-Free Methods

The scaffold-free approach relies on cancer cells’ ability to self-assemble into 3D structures. These methods offer a simpler, more physiologically relevant way to model the TME by depending on cell–cell and cell–matrix interactions to form spheroids. Several techniques are commonly used in scaffold-free methods, each with specific advantages for studying tumor biology and drug resistance. The hanging drop technique, where cells are suspended in culture medium droplets to aggregate and form spheroids, is widely used [29]. This method produces spheroids with uniform sizes and shapes, making it ideal for drug screening and quantitative analyses. Similarly, the liquid overlay technique [30] uses non-adherent surfaces, like hydrophobic polymer- or agarose-coated plates, to prevent cell attachment and encourage spheroid formation. Microfluidic devices and bioreactors, such as rotary cell culture systems, have also been explored to generate more physiologically relevant spheroids [31,32]. These scaffold-free methods provide valuable insights into the GSC niche by replicating the 3D structure and cellular interactions within the TME.

#### 3.1.1. Hanging Drop Method

The hanging drop method is a simple, cost-effective technique that produces highly uniform spheroids. It involves suspending a small volume of cell suspension as a drop on the lid of a culture dish, allowing cells to aggregate at the liquid–air interface due to gravity [33]. Generally, GBM spheroids, regardless of their formation method, display heightened drug resistance and unique gene expression profiles compared to 2D cultures, rendering them a valuable model for investigating tumor biology and therapeutic responses [34].

In early applications of the hanging drop method to construct GBM spheroids, readily available hydrophobic surfaces like culture dish lids or parafilm were commonly used. For example, Del Duca et al. employed the hanging drop technique to develop a reproducible method for generating implantable spheroids from murine and human brain tumor cell lines [35]. In their approach, 20 µL drops containing predetermined cell concentrations were suspended from culture dish lids, allowing cells to aggregate. These aggregates were transferred to agar-coated dishes to form three-dimensional spheroids, which were then implanted into collagen I gels to assess invasive activity. This hanging drop method effectively produced implantable spheroids with sustained invasion across all tested cell lines.

Recently, the GSC niche in GBM spheroids formed by the hanging drop method has been further explored. It was reported by Nusblat et al. that HIF-2α silencing in GSCs led to significant phenotypic changes, as assessed through cell migration assays, viability measurements, and immunofluorescence staining [36]. Specifically, HIF-2α suppression decreased GSC chemoresistance and migration activity. This study observed changes in stem and differentiation markers, with reduced expression of stem cell markers nestin and CD133 and increased expression of differentiation markers GFAP, β-tubulin, and MBP in GSC neurospheres. These findings indicated that HIF-2α inhibition reduced GSC stemness, promoted differentiation, and enhanced the efficacy of temozolomide.

**Figure 3:**
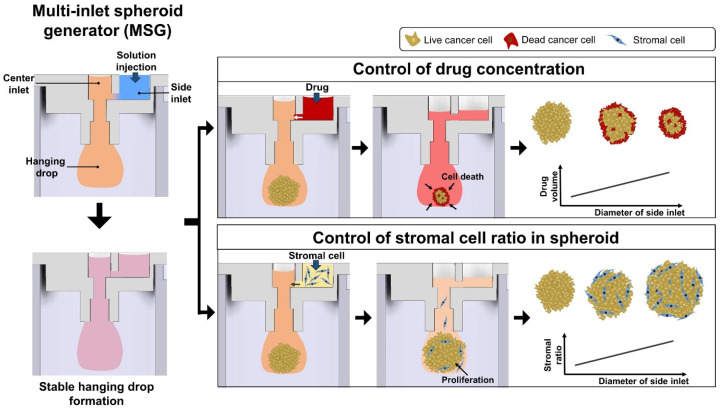
Multi-inlet spheroid generator (MSG) for tumor spheroid formation and modulation. The MSG allows additional solutions to be introduced through a side inlet without increasing force on the hanging drop, ensuring stability. This setup enables controlled addition of drugs or stromal cells, providing precise control over drug concentrations and stromal cell ratios. The MSG is, thus, ideal for high-throughput screening and accurate modeling of the tumor microenvironment [37].

Despite the simplicity and low cost of the hanging drop method, it has limitations, particularly its susceptibility to droplet detachment. This issue becomes more significant when adding liquids like drugs or stromal/immune cells directly to the spheroid-containing drop, complicating high-throughput screening and the inclusion of stromal cells to mimic the TME. To address these challenges, advanced hanging drop methods have been developed. We, for instance, introduced a multi-inlet spheroid generator (MSG), improving upon the traditional single-inlet design [37] (Figure 3). The MSG features two inlets—a center and a side inlet—allowing for additional solutions without increasing the force on the hanging drop. This design enables larger liquid volumes, reducing the risk of spheroid detachment during pipetting. Additionally, the diameter of the side inlet can be adjusted, and increasing the diameter enhances the liquid-holding capacity of the MSG during secondary additions, allowing for higher stromal cell ratios or drug concentrations to be introduced, enabling more precise control of the tumor–stroma ratio and drug concentration within the spheroid. This makes it possible to more efficiently construct complex GSC niches. As a result, it provides greater flexibility for high-throughput drug screening and the production of varied spheroids, making it ideal for studying intricate tumor microenvironments. Chen et al. developed a microfluidic filter plate based on the hanging drop method to facilitate spheroid formation while separating unbound or dead cells during cytotoxicity assays [38,39]. This method enables direct optical imaging to measure drug-induced cytotoxic effects on tumor spheroids, eliminating the need for live or dead fluorescent staining. It offers a cost-effective way to evaluate T-cell cytotoxicity with chimeric antigen receptors, enhancing immune cell-based assays and drug testing in 3D tumor models. Additionally, Tang et al. reported a method for producing heterocellular spheroids with controllable core–shell structures using inertial focusing in rotating hanging droplets [40]. These core–shell models exhibit biological functions significantly different from conventional heterocellular models, emphasizing the role of spatial arrangement in tissue function. This technique may allow for precise control over GBM spheroid size and geometry by adjusting cell suspension density and droplet morphology. Further advances, such as incorporating microfluidics, offer the potential to overcome the limitations of traditional methods and enable more sophisticated recapitulation of the GSC niche.

#### 3.1.2. Low-Adhesion Plates

Low-adhesion plates are designed to prevent cell attachment, promoting cell aggregation and spheroid formation. This method is widely used for its simplicity and scalability, facilitating high-throughput drug screening [41]. Tumor spheroids formed in these plates exhibit more physiologically relevant cell–cell interactions and extracellular matrix (ECM) production, making them valuable for investigating drug resistance and testing potential therapeutics [42]. For example, studies have shown that growing GBM spheroids under hypoxic conditions can induce a shift to a more glycolytic metabolism, a hallmark of GSCs [43]. Similarly, ultra-low-adhesion hydrogels, such as N-octanoyl glycol chitosan, have been developed to generate GBM spheroids as in vitro tumor models [44].

**Figure 4:**
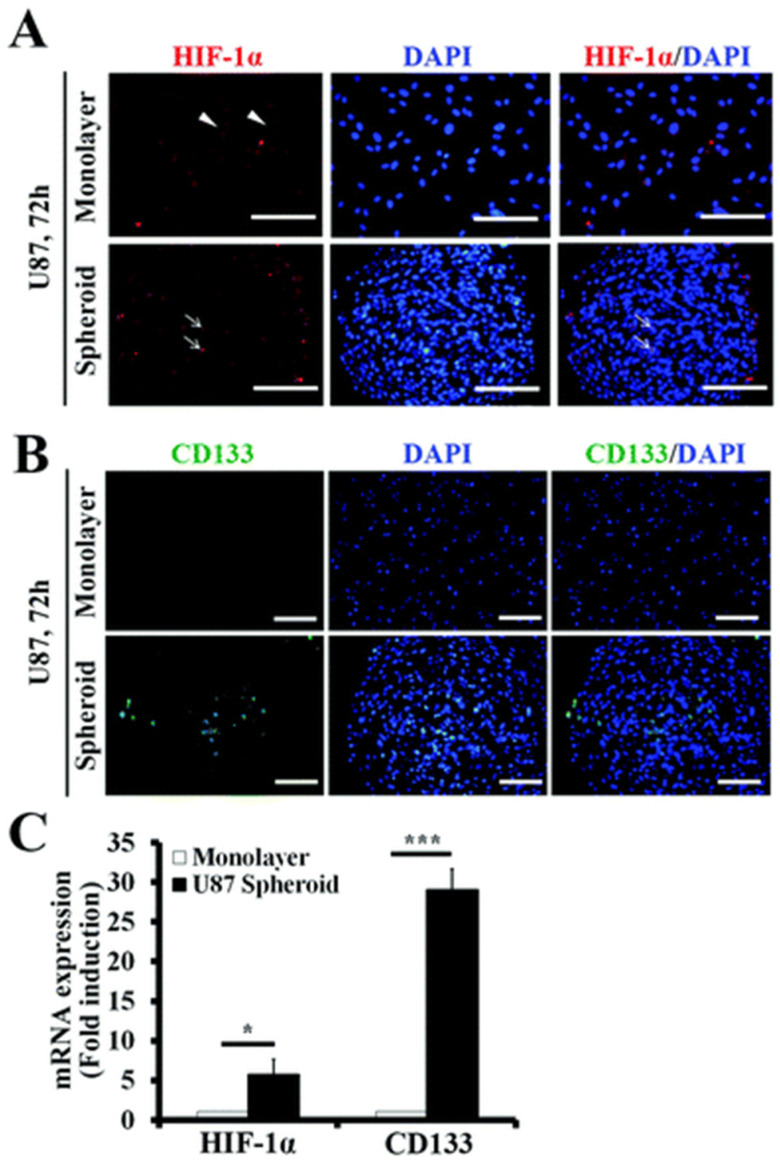
Expression of GSC markers HIF-1α and CD133 in U87 cell monolayers and spheroids formed in wells and ULA plates. (**A**) HIF-1α is absent in the nucleus of monolayer cells but localized in the nuclei of cells within spheroids. Scale bar: 100 μm. (**B**) CD133 expression is higher in spheroids compared to monolayers, indicating an increased stem cell population. Scale bar: 100 μm. (**C**) qRT-PCR analysis reveals elevated mRNA levels of HIF-1α and CD133 in spheroids, confirming hypoxic conditions and enhanced stemness [45]. Student’s *t*-test, * *p* < 0.05, ** *p* < 0.01, *** *p* < 0.001. Reprinted under the terms of Creative Commons Attribution 4.0 International (CC BY 4.0) license.

One of the most commonly used tools is the ultralow attachment (ULA) 96-well plate, which allows for easy manipulation. However, these plates can produce spheroids of varying sizes, as some may adhere to the well walls or multiple spheroids may form in a single well. Despite this, spheroids cultured in ULA plates retain their phenotypic traits, and uniform-sized spheroids can also be achieved by adjusting parameters like the center-to-center distance between polydimethylsiloxane (PDMS) micropillars [45]. Using this method, we generated a uniform population of GBM spheroids with an average diameter of 200 μm. These spheroids exhibited significantly higher expression of GSC markers, such as hypoxia-inducible factor-1α (HIF-1α) and CD133 [46], compared to GBM cells cultured in a monolayer (Figure 4).

Spheroids can be used not only for brain tumor research, but also for studying the permeability of substances across the blood–brain barrier (BBB) in normal physiology. BBB prevents blood-borne substances from entering the central nervous system. Isogai R et al. performed the permeability evaluation of macromolecules in multicellular spheroidal BBB models formed by using 96-well V-bottom plates [46].

The platform’s simplicity and compatibility with microfluidic components, such as channels and medium reservoirs, make it suitable for high-throughput screening. Mathew-Schmitt S et al. established a human blood–tumor barrier (BTB) in vitro test system for therapeutic screening [47]. GBM spheroids generated by seeding the glioblastoma cell lines in 24-well AggreWell™ 400 plates were co-cultured with human induced pluripotent stem cell (hiPSC)-derived brain capillary endothelial-like cells (iBCECs) in a cell culture insert-based format. Preclinical therapeutic screening and GBM-induced pathological changes at the BBB were tested in the BTB in vitro test system. Recently, Park et al. introduced a novel spheroid culture system that integrates a mesh structure coated with hexanoyl glycol chitosan, an ultralow adhesion material, into culture dishes to enhance ovarian spheroid formation [48]. This approach could enable precise control of GBM spheroid generation without traditional molding processes. Despite the limitations of low-adhesion plates in fully replicating the tumor microenvironment due to the absence of ECM, their utility in supporting high-throughput screening makes them essential for preliminary drug testing and functional assays, particularly in cancer stem cell research.

#### 3.1.3. Magnetic Levitation Method

Magnetic levitation is a technique that uses magnetic fields to suspend cells without physical contact. Pioneered by Dr. Utkan Demirci’s team, this method embeds cells with magnetic nanoparticles and uses magnetic fields to levitate and aggregate them into spheroids [49]. This innovative approach enables the creation of 3D cell cultures for investigating biological processes and conducting high-throughput cancer research screening [50,51]. Human GBM cells cultured using magnetic levitation showed both morphological and molecular similarities to human tumor xenografts in immunodeficient mice. Notably, N-cadherin, a transmembrane protein involved in cell–cell adhesion, was expressed in the membrane, cytoplasm, and cell junctions of 3D-levitated cells, mimicking its expression in tumor xenografts. In contrast, 2D cultures showed N-cadherin only in the cytoplasm and nucleus, with no membrane expression. Magnetic levitation allows for the formation of large, homogeneous spheroids of other type of cancer cells, including breast cancer, without the need for scaffolding materials [52].

**Figure 5:**
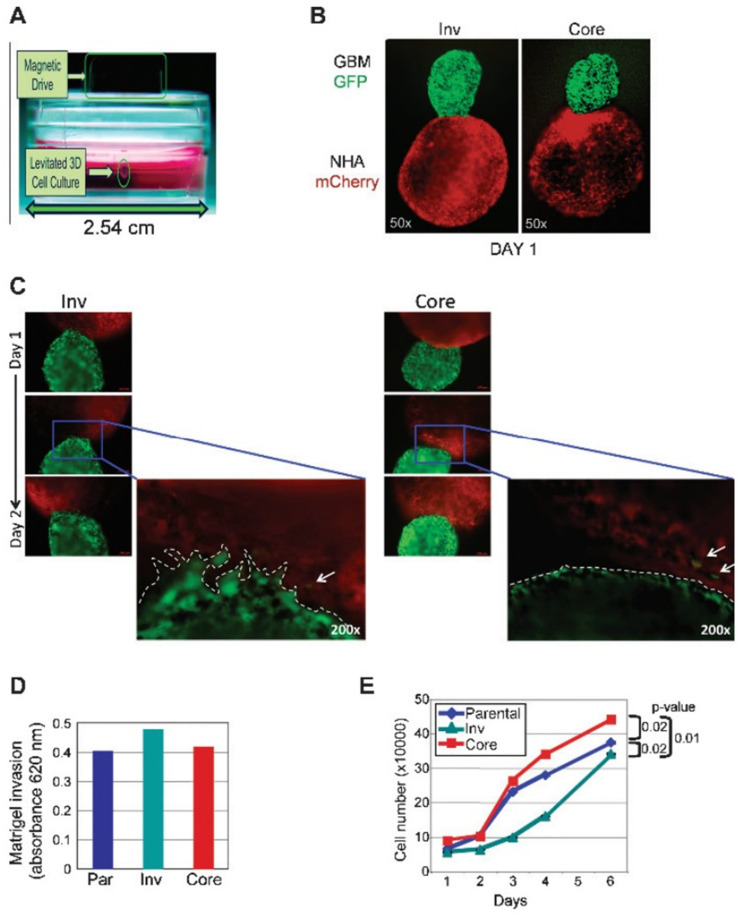
In vivo-selected Inv cells show increased invasion and reduced proliferation compared to Core cells. (**A**) In the 3D invasion assay setup, GBM or normal human astrocyte (NHA) cells treated with Au-Phage-FeO were suspended by a magnetic field. (**B**) GFP-labeled Inv and Core cell spheroids and mCherry-labeled NHA spheroids were formed separately and then magnetically guided together. (**C**) Serial fluorescence imaging over 48 h revealed an invading front of Inv cells at the contact area with NHAs (dotted line), while Core cells maintained an intact surface with NHAs. (**D**) Matrigel invasion assay confirmed higher invasiveness in Inv cells compared to parental (Par) or Core cells. (**E**) MTT assay indicated that Core cells proliferated faster than Inv cells [53]. (Reprinted under the terms of Creative Commons Attribution 4.0 International (CC BY 4.0) license).

Magnetic levitation can be used to study spheroid formation, as well as in vitro invasion assays. Molina et al. used the method to form spheroids from matched sets of tumor mass (Core) and invasive (Inv) cells isolated from mouse brains (Figure 5A) [53]. Inv-GFP and Core-GFP spheroids were brought into contact with normal human astrocyte (NHA)-mCherry spheroids using a magnetic field (Figure 5B) and imaged over time (Figure 5C). While Core cell spheroids maintained intact edges with minimal invasion into the astrocyte structure (Figure 5C, right panel), Inv cell spheroids displayed edge breaching and increased invasiveness. The Matrigel transwell assay further confirmed a slightly higher invasive capacity of Inv cells compared to Core or parental cells (Figure 5D). Additionally, Inv cells exhibited significantly reduced proliferation compared to Core cells (Figure 5E), indicating a trade-off between invasiveness and proliferation. These results demonstrate that magnetic levitation is a highly effective method for studying the invasive properties of GBM cells within brain parenchymal models.

### 3.2. Scaffold-Based Methods

While scaffold-free methods have provided insights into GBM pathology, they lack the full 3D ECM microenvironment of native tissue. To address this, researchers have focused on modeling invasion using 3D in vitro culture models, embedding tumor spheroids within 3D hydrogels composed of tissue ECM preparations or purified ECM proteins like collagen I and employing 3D bioprinting [54,55,56].

#### 3.2.1. Embedding GBM Spheroids in ECM Gels

**Figure 6:**
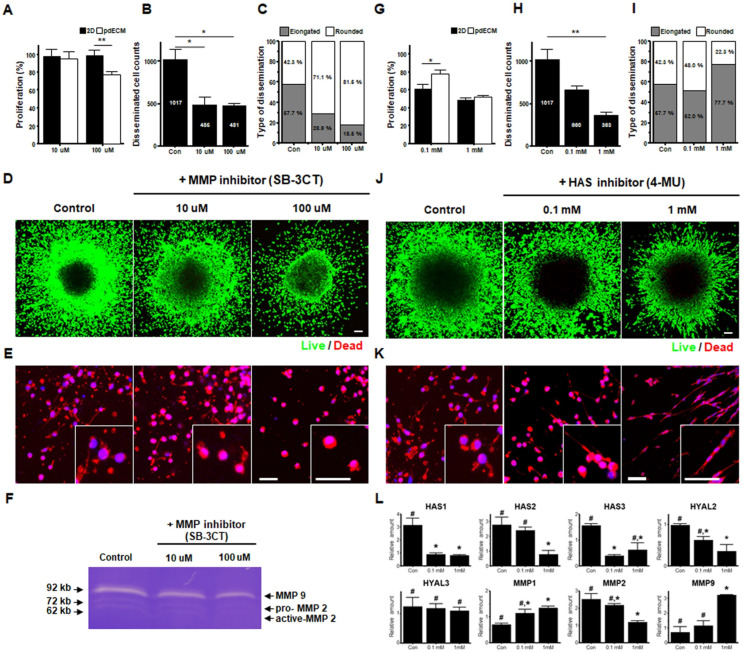
The impact of the ECM microenvironment on the responsiveness of patient-derived glioblastoma cells (pdGCs) to various inhibitors. (**A**) Proliferation of pdGCs in 2D and pdECM environments with SB-3CT (10 µM, 100 µM). Decreased proliferation in pdECM at 100 µM. Student *t*-test, * *p* < 0.05; ** *p* < 0.01. (**B**,**D**) The MMP inhibitor SB-3CT inhibits pdGC invasion dose-dependently. ANOVA, * *p* < 0.05. Scale bar = 100 μm. (**C**,**E**) SB-3CT increases the proportion of rounded invading cells (71.1% at 10 µM, 81.5% at 100 µM). (**F**) Zymography showing downregulation of MMP2 and MMP9 at higher SB-3CT doses. (**G**) Proliferation of pdGCs after the hyaluronan synthase (HAS) inhibitor 4-MU treatment (0.1 mM, 1 mM) in pdECM and 2D. Student *t*-test, * *p* < 0.05. (**H**,**I**) 4-MU reduces invasion and causes a morphological shift from rounded to elongated cells in pdECM. ANOVA, ** *p* < 0.01 (**J**) Representative image of 4-MU treated pdGCs in pdECM hydrogels after 72 h invasion. (**K**) Magnified images showing elongated cell morphology after 4-MU treatment. Scale bar = 100 μm. (**L**) Molecular profiling showing downregulation of HAS1, HAS2, HAS3, MMP2, and hyaluronidases 2 (Hyal2), with increased MMP1 and MMP9 after 4-MU treatment [57]. Different letters indicate a significant difference statistically by ANOVA, *p* < 0.05 (Reprinted under the terms of Creative Commons Attribution 4.0 International (CC BY 4.0) license).

Scaffold-based approaches in biofabrication use biomaterials to create a supportive framework that mimics the ECM of native tissues, enabling cell growth, organization, proliferation, differentiation, and function in a controlled environment. Hydrogels, hydrophilic polymer networks capable of retaining large amounts of water, are commonly used as scaffolds due to their biocompatibility and tunable physical properties. These hydrogels can be engineered to replicate the mechanical properties of brain tissue, providing a suitable environment for glioblastoma cells to grow and interact. For instance, Koh et al. developed a brain-derived extracellular matrix hydrogel to support the encapsulation and culture of patient-derived glioblastoma cells in 3D [57]. Notably, after inhibiting ECM remodeling enzymes like matrix metalloproteinases (MMP) 2/9 and hyaluronan synthase (HAS), the GBM cells underwent morphological changes and exhibited reduced invasion (Figure 6). The hydrogel’s composition and stiffness were optimized to promote the formation of spheroids with enhanced stemness properties, including CD133 expression [58]. In addition to mimicking mechanical cues, hydrogels can be designed to incorporate bioactive signals like growth factors and cell adhesion peptides to further simulate the in vivo tumor microenvironment [59,60].

While these simplified paradigms have provided novel insights into GBM pathology, they lack the full 3D ECM microenvironment of native tissue. To address this, researchers have focused on modeling invasion using 3D in vitro culture models, embedding tumor spheroids within 3D hydrogels made from tissue ECM preparations or purified ECM proteins like collagen I [54,55,56]. Hydrogels can encapsulate and gradually release growth factors and drugs, enhancing cancer stem cell proliferation and differentiation [61]. For instance, hyaluronic acid matrices, abundant in the brain, successfully recapitulated TGF-β-induced invasion, offering a valuable platform for further study. This research showed that GSC invasion of HA matrices could be predicted by TGF-β receptor 2 expression and SMAD2 phosphorylation, suggesting a specific pathway through which TGF-β influences GSC invasion. Additionally, GSC spheroid invasion strongly depends on the presence of RGD peptides on the HA backbone. Mimicking the ECM is crucial for studying cancer stem cell behavior in conditions similar to their natural environment. Studies show that hydrogels promote tumor spheroid formation, better replicating tumor architecture and the microenvironment compared to 2D cultures [44].

In summary, scaffold-based 3D culture systems have emerged as powerful tools for replicating the GBM tumor microenvironment and studying GSC behavior.

#### 3.2.2. 3D Bioprinting

Three-dimensional bioprinting is an advanced scaffold-based biofabrication technique that involves the precise layering of bioinks—composed of living cells and biomaterials—to create complex, three-dimensional tissue structures [62]. This technology mimics the natural architecture of tissues by enabling controlled deposition of cells and ECM components, facilitating the formation of functional tissue models. Using computer-aided design, 3D bioprinting constructs biological structures layer by layer, offering unparalleled control over cell placement, matrix composition, and spatial organization [63]. Brain tumor models created through 3D bioprinting have been shown to more accurately recapitulate in vivo tumor characteristics compared to traditional 2D and 3D culture methods.

Several methods are used in 3D bioprinting [64]. Extrusion-based bioprinting continuously extrudes bioinks through a nozzle, allowing for the creation of large, complex structures [65]. This method is ideal for printing tissues that require high mechanical strength, such as cartilage or bone. Inkjet bioprinting, by contrast, deposits droplets of bioink onto a substrate through thermal, piezoelectric, or electromagnetic forces, making it suitable for high-resolution printing and constructing tissues with detailed microarchitectures [66]. Laser-assisted bioprinting uses laser pulses to precisely deposit cells and bioink onto a substrate, offering the fine control needed to build tissues with intricate structures [67].

In GBM research, 3D bioprinting enables the creation of highly customized tumor models that replicate key features of the in vivo tumor microenvironment, including heterogeneous cell populations, ECM composition, mechanical properties, and vascular networks [64,68]. By incorporating various cell types such as GSCs and differentiated glioma cells, along with ECM components, in a spatially organized manner, 3D bioprinting offers a more accurate platform for studying GBM biology, invasion mechanisms, and drug resistance.

Various biomaterials, such as gelatin, alginate, and collagen, are extensively utilized in 3D bioprinting to offer structural support and bioactive cues crucial for GBM cell growth, migration, and differentiation [69]. These materials are selected for their biocompatibility, mechanical properties, and capacity to support glioblastoma cell adhesion and proliferation. Gelatin, derived from collagen, provides a natural environment that fosters the adhesion and survival of GSCs [70]. Alginate, a polysaccharide, forms hydrogels with adjustable mechanical properties that can replicate brain tissue stiffness, making it ideal for cultivating GBM cells [71]. Collagen, the most abundant protein in the brain’s ECM, offers structural integrity and enhances GBM cell adhesion, migration, and differentiation [72]. By integrating these biomaterials with relevant cell types, growth factors, and other ECM components, researchers can develop GBM models that closely mimic the in vivo tumor microenvironment.

**Figure 7:**
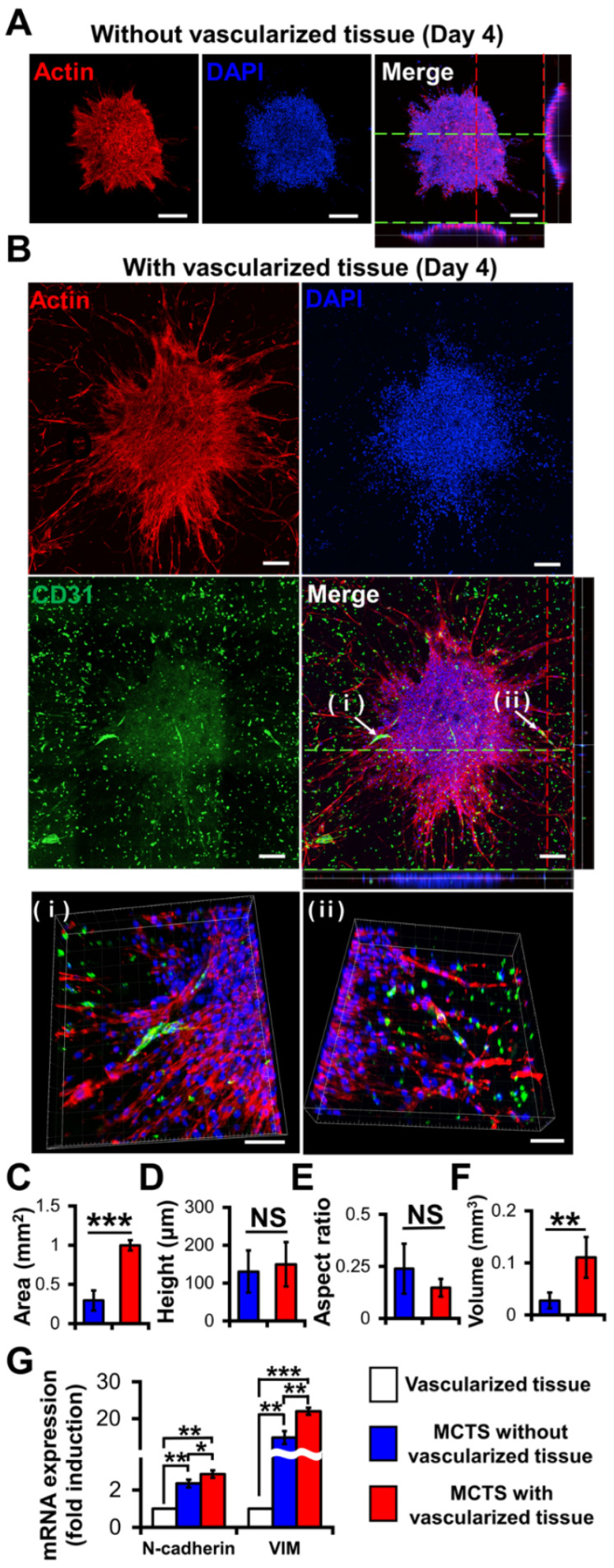
The effects of vascularized tissue on the growth and epithelial-to-mesenchymal transition (EMT) of GBM multicellular tumor spheroids (MCTSs). The figure compares multicellular GBM spheroids without (**A**) and with (**B**) vascularized tissue. The scale bar represents 200 μm or ((**B**) (i,ii)) 100 μm. GBM spheroids were fixed and stained for CD31 (green) or F-actin (red). CD31, an endothelial marker, showed that vascularized structures infiltrated into MCTSs (**B**(**i**,**ii**)). Nuclei were stained with DAPI (blue). Measurements included tumor area (**C**), height (**D**), aspect ratio (**E**), and volume (**F**) for spheroids with and without vascularization. (**G**) Additionally, mRNA expression levels of N-cadherin and vimentin were analyzed [73]. Student’s *t*-test; * *p* < 0.05, ** *p* < 0.01, *** *p* < 0.001; “NS” denotes “not significant”. (Reprinted under the terms of Creative Commons Attribution 4.0 International (CC BY 4.0) license).

One of the major challenges in constructing GBM models is fabricating vascularized structures, which are essential for providing nutrients and oxygen to cells, ensuring their long-term viability and function. Advancements in bioprinting techniques, such as incorporating endothelial cells and growth factors, are being explored to address this issue. We reported the integration of vascular networks within 3D GBM models to better study GBM cell–endothelial cell interactions, angiogenesis, and the effects of anti-angiogenic therapies (Figure 7) [73]. Uniform-sized GBM multicellular tumor spheroids (MCTSs) were seeded onto the GAF hydrogel layers with and without vascularized tissues. It was observed that CD31, an endothelial marker, indicated vascular structures infiltrating into MCTSs (Figure 7B(i,ii)). Additionally, MCTSs with vascularized structures, composed of U87 cells, HUVECs, and fibroblasts, exhibited significantly higher expression of epithelial-to-mesenchymal transition (EMT) markers, N-cadherin and vimentin (VIM), compared to non-vascularized MCTSs (Figure 7G). This increase in EMT marker expression, linked to angiogenesis and invasion, suggests that vascularization promotes more aggressive tumor behavior.

Despite the challenges, 3D bioprinted GBM models have provided valuable insights into the interactions between GSCs and the tumor microenvironment, revealing how these interactions contribute to drug resistance and tumor progression. Additionally, they have been used to screen potential therapeutic agents, offering a more predictive platform for evaluating drug efficacy and safety.

In summary, scaffold-based approaches in biofabrication, particularly 3D bioprinting, provide a powerful tool for creating realistic tumor models that allow for the study of complex interactions between cancer cells and their microenvironment. These models hold great potential for advancing cancer research, especially in understanding drug resistance and developing more effective treatments. By addressing current challenges and continuing innovation, scaffold-based biofabrication can significantly impact oncology and personalized medicine.

### 3.3. Organoid Culture

GBM organoids are 3D in vitro models that closely mimic the architecture, cellular diversity, and microenvironment of GBM tumors [74,75,76]. Derived from patient tumor samples or stem cells, these organoids are cultured to form self-organized, tissue-like structures. GBM organoids replicate key features of primary patient tumors, including the presence of GSCs, tumor heterogeneity, and complex cellular interactions within the tumor microenvironment. These models are especially valuable for studying GBM biology and drug responses in a more physiologically relevant context than traditional 2D cultures [77].

**Figure 8:**
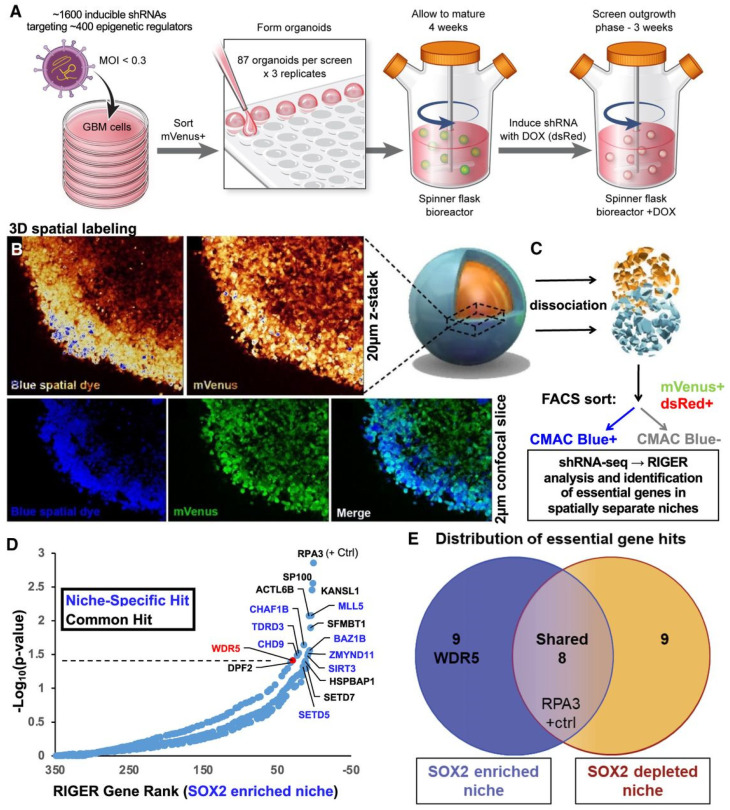
A spatial functional genomics screening process to identify essential genes in GSC niches using organoid models. (**A**) GBM GSCs were infected with an inducible shRNA library targeting epigenetic modifiers and grown into organoids. Upon doxycycline induction, shRNA expression (indicated by the dsRed reporter) was activated, and organoids were labeled with a blue dye (CMAC) to mark the outer rim. (**B**) Z-stack images and individual slices show labeling intensity in GBM528 organoids, highlighting overlap between labeled regions. (**C**) Organoids were dissociated, and single cells were sorted into rim (CMAC^+^) and core (CMAC^−^) populations using FACS. DNA was then extracted for barcode sequencing to analyze shRNA distribution. (**D**) A rank-ordered list of genes targeted by the shRNA screen shows depletion in the SOX2-enriched niche (CMAC^+^), highlighting niche-specific hits (blue) and common hits (black). (**E**) A Venn diagram displays genes specific to SOX2-enriched and SOX2-depleted niches, along with those common to both regions [79]. (Reprinted under the terms of Creative Commons Attribution 4.0 International (CC BY 4.0) license).

GBM organoids are typically generated by culturing tumor or stem cells in a 3D matrix, often using a scaffold-free method or embedding them in hydrogels like Matrigel, which provides essential ECM components for supporting cell growth and organization. Cells are seeded to promote self-assembly, forming spherical structures that can be maintained long-term. The culture medium is supplemented with growth factors and nutrients to support GBM cell development and proliferation, allowing the organoids to form complex structures resembling primary tumors. By using patient-derived GBM cells, organoids retain the genetic and epigenetic characteristics of the original tumor, making them a robust model for personalized medicine and for studying tumor behavior, invasion, and therapeutic resistance [77,78]. These organoids facilitate interactions between cells in different states within an in vitro system, effectively mimicking the tumor microenvironment. GBM organoid models have been used to replicate cellular states found in primary patient tumors, including specific SOX2^+^ GSC niches [79]. The outer rim of the GBM organoids displays high proliferative activity and is enriched with SOX2^+^ cells, which contribute to resistance against standard-of-care treatments and other clinically relevant therapies (Figure 8). A spatially resolved loss-of-function screen in the organoids revealed that WDR5 is indispensable for maintaining the SOX2-enriched, therapy-resistant niche.

### 3.4. Microfluidic Device

Microfluidics is a technology that manipulates small fluid volumes in microscale channels, creating controlled environments that mimic in vivo conditions [80]. This technology is crucial in cancer research, especially for GBM, an aggressive brain cancer known for its rapid progression and recurrence. Microfluidic models significantly advance the replication of the complex TME of GBM, which includes various cancerous and non-cancerous cells, biomolecules, and ECM components that drive tumor growth [81].

A key material in constructing microfluidic devices is polydimethylsiloxane (PDMS), valued for its transparency, ease of fabrication, and biocompatibility [82]. PDMS allows for precise control over nutrient and oxygen gradients, making it ideal for studying complex tumor behaviors, including those seen in GBM. Its versatility enables researchers to closely mimic the physical and chemical conditions of the TME, providing valuable insights into tumor dynamics and therapeutic responses [83].

Microfluidic platforms are essential for modeling GBM, as they can accurately replicate the complex environment of the BBB, crucial for understanding GSCs. The BBB is a major obstacle in GBM treatment, limiting therapeutic agent delivery to the tumor [84]. For example, the microfluidic system models the selective permeability of the BBB by layering human brain endothelial cells (HBMEC) and pericytes (HBVP). The upper layer mimics the endothelial barrier, while the lower layer stabilizes barrier function through pericyte interactions. A porous membrane between the layers allows for controlled diffusion of substances, replicating selective transport across the BBB. Physiological shear stress mimics blood flow conditions, further enhancing the accuracy of the model [85]. Microfluidics allows for precise modeling of how drugs, including nanoparticles, penetrate the BBB and reach GBM tumors, while enabling real-time, high-resolution monitoring of drug delivery and GSC interactions. By simulating in vivo-like conditions, such as hypoxic tumor regions, microfluidics enhances preclinical testing of targeted therapies and improves the predictability of treatment outcomes. These platforms are invaluable for studying GSC behavior and evaluating therapies in a realistic, controlled environment [86].

Among scaffold-based methods, microfluidics excels in precisely creating vascular structures, providing fluid flow, and observing GBM-induced angiogenesis [87]. This leads to the development of a 3D organotypic microfluidic model incorporating a pre-established microvascular network. This innovative model allows for the investigation of interactions between patient-derived GSCs and endothelial cells, focusing on tumor invasion. The pre-established vasculature enhances GSC invasion and promotes an invasive morphology while maintaining the stem-like characteristics of the GSCs. Notably, CXCL12-CXCR4 signaling was identified as a key pathway driving GSC invasion, and the CXCR4 antagonist AMD3100 successfully reduced invasion in co-culture conditions, highlighting the importance of studying multi-cell interactions for drug discovery [88].

**Figure 9:**
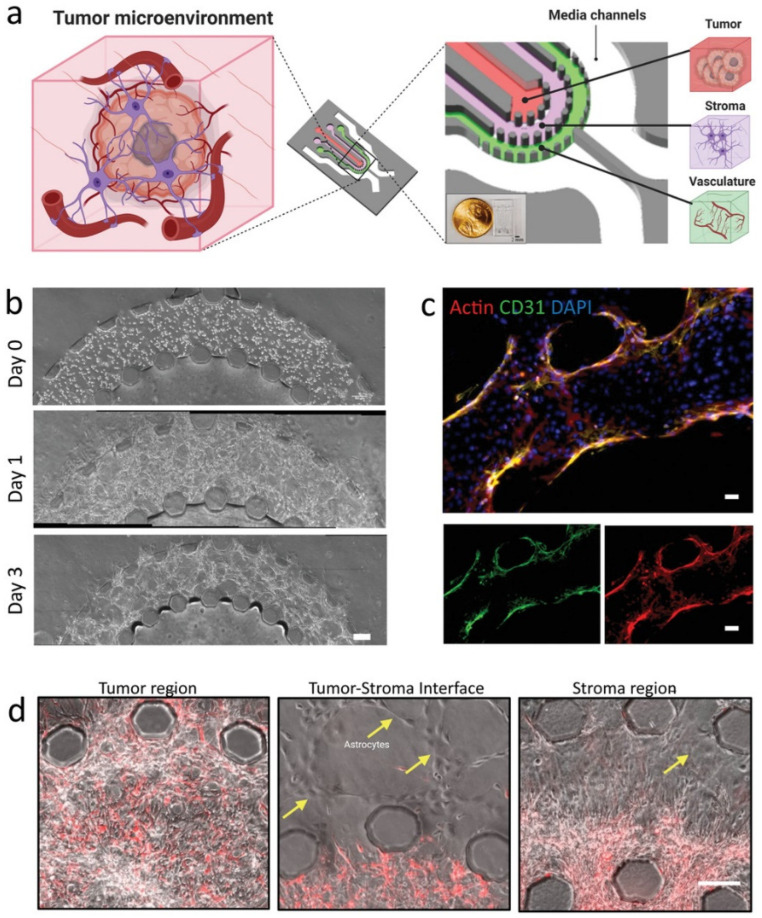
Microfluidic model of GBM tumor microenvironment. (**a**) Schematic of the microfluidic device showing compartments for tumor, stroma, and vasculature. The model comprises three concentric cell culture regions—vasculature, stroma, and tumor—surrounded by media channels. Hexagonal microposts, spaced evenly at 100 µm, define these regions, allowing clear distinction between tumor, stroma, and vascular components in an organotypic arrangement while maintaining interconnectivity. This setup enables cellular interactions and the invasion of GSCs from the tumor region into the adjacent stromal region. (**b**) Phase-contrast images of cell organization at Day 0, Day 1, and Day 3, illustrating structural development within the device. Scale bar: 100 μm. (**c**) Immunofluorescence staining for actin, CD31, and DAPI at the tumor–stroma interface, demonstrating endothelial network formation. Scale bar: 50 μm. (**d**) High-magnification images of the tumor region, tumor–stroma interface, and stroma region, with astrocytes indicated by yellow arrows. Scale bar: 20 μm [91]. (Reprinted under the terms of Creative Commons Attribution 4.0 International (CC BY 4.0) license).

Recent advancements in microfluidic models have improved the replication of the GBM TME, particularly the perivascular niche, which is crucial for supporting GSCs [89,90]. These 3D models more accurately represent cellular interactions between GSCs, endothelial cells, and astrocytes, demonstrating enhanced GSC invasion and the maintenance of stem-like properties. For example, Adjei-Sowah, E.A. et al. developed a microfluidic GBM tumor-on-a-chip model with three interconnected regions—vasculature, stroma, and tumor (Figure 9) [91]. Hexagonal microposts, spaced 100 µm apart, separated these regions while preserving their distinct characteristics, enabling cellular interactions and GSC invasion from the tumor to the stroma. Using this model, they performed single-cell RNA sequencing (scRNA-seq) to reveal key ligand–receptor pairs, such as SAA1-FPR1 and RSPO3-LGR6, driving GSC migration. This approach highlights the potential of microfluidic models for uncovering molecular mechanisms and advancing drug discovery in GBM, with future studies possibly integrating immune cells to explore their role in chemo-resistance and immunosuppression.

Microfluidic devices provide a valuable platform for mimicking the glioblastoma TME, but they face several limitations. Most 3D printers used for these devices cannot achieve resolutions finer than 200 μm, which can affect the structural integrity of small or complex channels, potentially causing flow disruptions or clogging [92]. Materials like acrylate and acrylonitrile butadiene styrene may absorb lipids and proteins, compromising channel stability and experimental results [89]. While PDMS facilitates short-term gas and nutrient exchange, it is inadequate for long-term studies [93]. Additionally, replicating the GBM niche and isolating GSCs in these systems remains challenging.

Despite these limitations, microfluidic devices offer significant advantages over traditional 2D and 3D cultures by simulating dynamic fluid flow as well as nutrient and oxygen gradients, which are crucial for accurately studying tumor growth, metastasis, and drug responses [94]. This precision enables modeling in vivo conditions like pseudopalisade formation and hypoxia, offering insights into key features of GBM progression [95]. To fully realize their potential, addressing these limitations through improvements in materials and design is essential, enhancing the physiological relevance and effectiveness of microfluidic platforms in cancer research and therapy development [96].

## 4. Current Challenges and Future Directions in GBM Tumor Modeling: Scaffold-Free, Scaffold-Based, Organoid, and Microfluidic Approaches

A significant challenge for scaffold-free methods is their inability to fully replicate the complex tumor microenvironment, particularly the ECM. Since these models rely on the natural self-aggregation of cells without external support, they often lack the structural and mechanical cues that influence cell behavior in vivo. Additionally, capturing tumor heterogeneity is difficult, as the model tends to be more simplistic than the actual diversity found in GBM tumors. Future directions for scaffold-free methods involve improving techniques to better mimic physiological conditions, such as introducing oxygen gradients or other environmental factors to enhance the realism of these models.

For scaffold-based methods, a primary challenge is selecting biomaterials that accurately represent the brain’s ECM while maintaining biocompatibility and mechanical properties conducive to cell growth. Although scaffold-based methods offer greater control over cell organization and microenvironmental conditions, replicating the full complexity of the brain’s structural and biochemical properties remains difficult. Additionally, fabricating models with vascular structures to support long-term cell viability is challenging. Future developments will likely focus on creating more sophisticated scaffolds that incorporate dynamic elements like nutrient delivery systems, vascularization, and real-time monitoring, bringing these models closer to mimicking the in vivo conditions of glioblastoma.

One key challenge in organoid culture methods is the uncontrollability of organoid size and morphology, leading to variability in experimental outcomes. While organoids excel at replicating the 3D architecture and cellular heterogeneity of tumors, their complexity makes it difficult to maintain consistent structural features across samples. Another challenge is the lack of vascularization, which limits their size and viability over extended periods. Future directions for organoid models will likely focus on developing more standardized protocols to reduce variability and enhance reproducibility. Advances in vascularization techniques and incorporating immune system components are also crucial for making organoids more reflective of the in vivo tumor environment.

Microfluidic models offer unique advantages in GBM research, enabling precise control over the TME and replicating features like the BBB for real-time studies on drug delivery and cellular interactions. However, limitations persist as the 3D printing resolution in microfluidics is often insufficient for small channels, and materials like acrylate and ABS can absorb proteins, affecting channel stability. Additionally, while PDMS allows for short-term gas and nutrient exchange, it is unsuitable for long-term studies due to its tendency to absorb small molecules and degrade mechanically, underscoring the need for improved materials and designs to fully harness the potential of microfluidic platforms in cancer research.

In summary, although each method has its strengths, specific challenges limit their ability to fully replicate the GBM tumor microenvironment, as summarized in Table 1. Future research will focus on addressing these limitations by introducing more physiologically relevant conditions and improving reproducibility and scalability, particularly in areas like vascularization and tumor heterogeneity.

## 5. Conclusions

Scaffold-free, scaffold-based, and microfluidics models, as well as GBM organoids, offer valuable in vitro platforms to recapitulate the complex GBM tumor microenvironment, especially the niches of GSCs. Scaffold-free methods provide simplicity and cost-effectiveness, but lack the structural complexity of the tumor’s ECM. Scaffold-based approaches, using hydrogels and other materials, better mimic the brain’s ECM and support cellular interactions and invasion studies, though they still face challenges in fully replicating tumor biology. GBM organoids, while effective at recapitulating the 3D architecture and cellular diversity of primary tumors, face limitations in consistency and vascularization. Microfluidic GBM models advance the replication of the complex TME of GBM, which includes various cancerous and non-cancerous cells, biomolecules, and ECM components that drive tumor growth.

Each model has distinct advantages and limitations, and further innovations in biofabrication techniques, such as incorporating vascular networks and immune system components, will be crucial for improving their physiological relevance. Moving forward, these models will play a vital role in developing more effective therapies targeting GSCs and their role in GBM progression and therapeutic resistance.

## Figures and Tables

**Table 1:** Advantages and limitations of tumor spheroid formation methods.

Method	Technique	Mechanism	Advantages	Limitations	Reference
Scaffold-free	Hanging Drop Method	Suspends small cell droplets; allows spheroid formation via gravity	Simple; cost-effective; uniform spheroids	Susceptible to droplet detachment; limited scalability	[33]
	Low-Adhesion Plates	Uses non-adherent surfaces to prevent cell attachment to promote aggregation into spheroids	Easy to use; suitable for high-throughput screening	Variability in spheroid size; lacks ECM	[41,42]
	Magnetic Levitation Method	Uses magnetic nanoparticles to levitate and aggregate cells into spheroids	Forms large spheroids rapidly	Costly; potential biocompatibility issues	[49,50]
Scaffold-based	ECM Gels	Embeds cells in hydrogels to mimic the natural tumor ECM microenvironment	High biocompatibility; mimics natural microenvironment	Limited mechanical strength; requires tuning of ECM	[57,59]
	3D Bioprinting	Layer-by-layer printing of bioinks to create complex 3D structures	Formation of functional tissue models; better mimics in vivo tumor traits	Expensive setup; limited bioink options	[62,63]
Organoids	GBM Organoids	Self-assembly of cells to create 3D tumor models	Mimics tumor heterogeneity and stem cell niches	Long culture times; variability in size and structure	[77]
Microfluidics	Microfluidic Devices	Uses microchannels to create controlled, dynamic environments for cell growth	Real-time monitoring; precise control of microenvironments	Complex fabrication; scalability challenges	[80,81]
